# Discrepancies in estimated glomerular filtration rate and albuminuria levels in ethnic minority groups – The multiethnic HELIUS cohort study

**DOI:** 10.1016/j.eclinm.2022.101324

**Published:** 2022-03-05

**Authors:** Brechje J.M.V. Huisman, Charles Agyemang, Bert-Jan H. van den Born, Ron J.G. Peters, Marieke B. Snijder, Liffert Vogt

**Affiliations:** a Department of Internal Medicine, Section Nephrology, Amsterdam UMC, University of Amsterdam, Meibergdreef 9, Amsterdam 1105 AZ, the Netherlands; b Department of Public and Occupational Health, Amsterdam UMC, University of Amsterdam Public Health Research Institute, Amsterdam, the Netherlands; c Department of Internal Medicine, Section Vascular Medicine, Amsterdam UMC, University of Amsterdam, Amsterdam, the Netherlands; d Department of Cardiology, Amsterdam UMC, University of Amsterdam, Amsterdam, the Netherlands; e Department of Clinical Epidemiology, Biostatistics and Bioinformatics, Amsterdam UMC, University of Amsterdam, Amsterdam Public Health Research Institute, Amsterdam, the Netherlands; f Amsterdam Cardiovascular Sciences, Amsterdam UMC, University of Amsterdam, Amsterdam, the Netherlands

**Keywords:** Estimated glomerular filtration rate, Albuminuria, Album-to-creatinine ratio, Multiethnic population, General population, Chronic kidney disease, HELIUS study

## Abstract

**Background:**

Classification of chronic kidney disease (CKD) and evaluation of prognosis is based on two components: estimated glomerular filtration rate (eGFR) and urinary albumin-to-creatinine ratio (ACR). In multiethnic populations, ethnic-specific discrepancies in both parameters may exist. It is unknown whether variations in CKD risk factors may explain these discrepancies.

**Methods:**

We cross-sectionally analyzed baseline eGFR (CKD-EPI formula) and ACR of 21,421 participants (aged 18–70 years) of the HELIUS cohort who were randomly sampled between 2011 and 2015, stratified by ethnicity, through the municipality register of Amsterdam. Six ethnic groups were distinguished, including participants of Dutch (4539), South-Asian Surinamese (3027), African Surinamese (4114), Ghanaian (2297), Turkish (3576) and Moroccan (3868) descent. Multiple regression analyses to determine ethnic differences were performed, with additional adjustments for age, sex, traditional cardiovascular and renal risk factors, and adjustment for level of education.

**Findings:**

Mean (SE) eGFR was higher in all ethnic minority groups as compared to Dutch participants (eGFR 94.7 ± 0.3 mL/min/1.73 m^2^) with age- and sex-adjusted differences ranging from 1.5 ± 0.30 in South-Asian Surinamese to 10.1 ± 0.28 mL/min/1.73 m^2^ in Moroccan participants. ACR was higher in ethnic minority groups as compared to Dutch participants (ACR 0.64 ± 0.20 mg/mmol), with age- and sex-adjusted differences ranging from 0.46 ± 0.20 in African Surinamese participants to 1.70 ± 0.21 mg/mmol in South-Asian Surinamese participants. Differences in both parameters diminished after multiple adjustments, but remained highly significant.

**Interpretation:**

Both eGFR and ACR are higher among ethnic minority groups as compared to individuals of Dutch origin—independent of age, sex, prevalence of traditional cardiovascular and renal risk factors, and parameters of socioeconomic status. Future studies should address the potential uncertainty in predicting CKD and CKD-related complications when using both parameters in ethnically diverse populations. Also, identification of driving factors leading to these discrepancies might contribute to improved population screening for CKD.

**Funding:**

The HELIUS study is conducted by the Amsterdam University Medical Center and the Public Health Service of Amsterdam. Both organizations provided core support for HELIUS. The HELIUS study is also funded by the Dutch Heart Foundation (2010T084), the Netherlands Organization for Health Research and Development (ZonMw: 200500003), the European Union (FP7: 278901), and the European Fund for the Integration of non-EU immigrants (EIF: 2013EIF013).

**Research in context**

**Evidence before this study**

Data from multiethnic populations indicate that differences in chronic kidney disease (CKD) prevalence and CKD risk exist with higher risk in ethnic minority groups. It is, however, unknown whether this is the result of discrepancies in estimated glomerular filtration rate (eGFR) and urinary albumin-to-creatinine ratio (ACR) between ethnic groups in an ethnically diverse population. Whether variations in CKD risk factors may explain discrepancies between ethnic groups is also unknown. We therefore searched Pubmed for publications in English with the following search “(((("Glomerular Filtration Rate"[Mesh] OR "Glomerular Filtration Rate"[Tiab] OR "eGFR"[tiab] OR "estimated glomerular filtration rate"[tiab]) OR ("Albuminuria"[Mesh] OR "Albuminuria"[tiab] OR "ACR"[tiab])) AND ((((("Racial Groups"[Mesh]) OR "Ethnic and Racial Minorities"[Mesh]) OR "Blacks"[Mesh]) OR "Minority Groups"[Mesh]) OR "Ethnicity"[Mesh] OR "Racial groups"[tiab] OR "Ethnic and racial minorities"[tiab] OR "Blacks"[tiab] OR "Minority groups"[tiab] OR "Ethnic*"[tiab])) AND ("CKD-EPI"[tiab])) AND ("Validation Study" [Publication Type])”. This search led to 12 results. However, none of the studies found in this search assessed discrepancies in age- and sex adjusted eGFR and/or ACR or reported between different groups of multiethnic populations.

**Added value of this study**

Our multiethnic cohort study, including large representative samples of six ethnic groups from Amsterdam, the Netherlands, shows that both eGFR and ACR are higher in ethnic groups of non-Dutch descent, also after multiple adjustments, including cardiovascular and CKD risk factors and a parameter of socioeconomic status.

**Implications of all the available evidence**

Adoption of the currently recommended approach for staging and predicting CKD and CKD-related complications, by using the composite of both eGFR and ACR, potentially leads to uncertainty in CKD prevalence and incorrect estimation of the risk of CKD-related complications in a multi-ethnic population–particularly, in ethnic groups that are recognized for a higher risk of CKD. In line with others, including the recent NKF-ASN taskforce, the data underscore the need to reassess the value of ethnic specific tools for predicting CKD risk and long-term CKD-related outcome.

Alt-text: Unlabelled box

## Introduction

Chronic kidney disease (CKD) is a growing major public health problem that has important impact both at the patient level, by lowering the quality of life and life expectancy, and at the population level by increasing health-care costs and the demand for health-care services.^1^^,^^2^ Even mildly impaired kidney function is associated with impaired health outcomes.^3^ Detection of CKD in earlier stages is therefore recommended in subjects at risk for CKD. According to the widely adopted Kidney Disease Improving Global Outcomes (KDIGO) guidelines, classification of CKD and evaluation of its prognosis is based on two renal components, represented by estimated glomerular filtration rate (eGFR) and albumin-to-creatinine ratio (ACR).^4^ Regular screening for both components is recommended in patients with diabetes mellitus, hypertension or with a history of cardiovascular disease (CVD) in most guidelines.^4^^,^^5^

In Europe and the US, not only the CKD prevalence is higher in ethnic minority groups of non-European descent, but CKD also progresses faster to ESRD.^6^, ^7^, ^8^, ^9^, ^10^ Whether this can fully be attributed to the higher prevalence of CKD risk factors is largely unknown. Moreover, it can be questioned whether the current screening tools for identification of CKD risk groups is applicable in all populations. The nowadays widely used creatinine-based CKD-EPI equation for eGFR makes distinction between African and European descent, but has limitedly been validated for other ethnic groups.^11^, ^12^, ^13^, ^14^, ^15^

Currently worldwide populations are becoming more ethnically diverse. In the Netherlands, 23% of the population has a migration background and is expected to increase to 39% by 2060.^16^ As a consequence, CKD prevalence is expected to rise in the upcoming years. Therefore, considering the increasing ethnic diversity of populations worldwide, identification of the driving factors that explain the CKD risk excess, as reflected by both eGFR and ACR, in multiethnic populations becomes highly relevant. In this large-scale, multiethnic population study, we aimed to assess discrepancies in both eGFR and ACR between six ethnic groups living in Amsterdam, the Netherlands, and to determine whether these differences could be attributed to presence of recognized CKD risk factors.

## Methods

### Study design

The HELIUS (Healthy Life in an Urban Setting) study, is a large-scale multiethnic cohort study conducted in Amsterdam, the Netherlands. Detailed information, the rationale, conceptual framework, design and methodology of HELIUS have been described in detail elsewhere.^17^^,^^18^ Briefly, the HELIUS study is a prospective population-based cohort study, that included the six largest ethnic groups living in Amsterdam, the Netherlands. HELIUS was set up to fill the gap in epidemiological health research on ethnic inequalities in health in Europe. The general objective of the HELIUS study is to unravel the unequal burden of diseases across these ethnic groups, with the main focus on cardiovascular diseases, mental health and infectious diseases. The overall aim is to provide knowledge for the improvement of healthcare and the prevention of communicable and non-communicable diseases. The HELIUS study was approved by the Institutional Review Board of Academic Medical Centre, at the University of Amsterdam (METC 10/100# 10.17.1729) and written informed consent was obtained from all participants.

### Study population

Between 2011 and 2015, persons aged 18–70 years were randomly sampled, stratified by ethnicity, through the municipality register of Amsterdam. The study included people of Dutch, South-Asian Surinamese, African Surinamese, Ghanaian, Turkish and Moroccan origin, all residents of Amsterdam. Suriname was a former Dutch colony. In 1975, during the process of decolonization, almost half of the entire Suriname population (including South-Asian, African and Javanese Surinamese) migrated to The Netherlands. South-Asian Surinamese living in the Netherlands originate from the Indian subcontinent and share a common ancestry with the South Asian Indian populations. African Surinamese share a common ancestry with the African-descent populations in the West. In the late 1960s and early 1970s, Turkish and Moroccan guest workers migrated to the Netherlands upon invitation by the Dutch government. From the second half of the 1970s onwards, these workers were joined by their families. From the 1980s, migration of Ghanaians to the Netherlands started. Ghanaian migrants are largely concentrated in the large cities in the Netherlands.^17^^,^^18^

For the current analyses, baseline data of 22,165 participants with data available on both questionnaire data and physical measurements were used. Participants with unknown ethnicity (*n* = 48), participants from Javanese Surinamese descent (*n* = 233) and participants with unknown Surinamese origin (*n* = 267) were excluded. Also, individuals without data regarding CKD (*n* = 122) and level of education (*n* = 195) were excluded. Therefore, analyses were conducted among 21,421 participants of Dutch (*n* = 4539), South-Asian Surinamese (*n* = 3027), African Surinamese (*n* = 4114), Ghanaian (*n* = 2297), Turkish (*n* = 3576) and Moroccan (*n* = 3868) origin.

### Data collection

Data was collected through questionnaire and physical examination, as well as biological samples, which were taken during study visits. All individuals selected to participate in the HELIUS study received a written invitation. The questionnaire addressed determinants and risk factors of cardiovascular disease. The potential for self-reporting bias is always a concern when using data from questionnaire with self-reported data. However, the HELIUS cohort study tried to reduce this to a minimum. Questionnaires were available in several languages, English for the Ghanaian participants and Turkish for Turkish participants. When participants were unable to complete the questionnaire themselves (due to language or reading problems) they were offered assistance from a trained ethnically matched same-sex interviewer, speaking their preferred language. Also, participants data are linked to registry data for routinely collect data on health outcomes and health care at the individual level. This has the advantage that clinical data (including self-reported data) can be compared with registry data (in case of self-reported diagnosis such as diabetes mellitus).

During the physical examination a fasting venous blood sample and morning urine sample were obtained. This was used to assess eGFR and ACR. No confirmation by a second assessment was performed. In total 90,019 persons were invited to participate in the HELIUS study, approximately 55% responded either by card or after a home visit by an ethnically matched interviewer. 24,789 subjects agreed to participation, for 22,165 of the participants, both questionnaires and physical examination were available for data analysis . The response rate, defined as the percentage of invited subjects from whom baseline data were obtained was 28% and varied between the ethnic subgroups (Dutch 33%, Surinamese 31%, Ghanaians 35%, Turks 22% and Moroccans 21%).^17^

### Measurements

#### eGFR and ACR

Serum creatinine concentration (in µmol/L) was determined by an enzymatic method (Roche C702 at the C8000 platform). Estimated glomerular filtration rate (eGFR) was calculated using the CKD-EPI creatinine equation, where we used the race correction factor for participants of African Surinamese and Ghanaian origin.^11^ An early morning urine sample was used for direct analysis of albuminuria and creatinine. Urinary albumin concentration (in mg/L) was measured by an immunochemical turbidimetric method (Roche Diagnostics). Urinary creatinine concentration (in mmol/L) was measured by a kinetic spectrophotometric method (Roche Diagnostics). Urinary albumin-creatinine ratio (ACR; expressed in mg/mmol) was calculated.

#### Kidney and CV risk factors

Body mass index (BMI) was calculated as weight (kg) divided by height squared (m^2^)—both measured in light clothing and without shoes to the nearest 0.1 kg and 0.1 cm, respectively. Type 2 diabetes was defined as a fasting glucose level >7 mmol/L or receiving glucose-lowering medication. Blood pressure (BP) was measured two times in a seated position, after the participant had been seated for at least 5 min. The mean BP of the two measurements was used. Hypertension was defined as a systolic BP >140 mmHg, or a diastolic BP >90 mmHg, or being on antihypertensive medication. Hypercholesterolemia was defined as total cholesterol >6.22 mmol/L. Plasma non-HDL cholesterol was calculated from plasma total cholesterol minus HDL-cholesterol. Smoking status was determined from the response to the question “Do you smoke at all?” and classified into smokers and non-smokers. Socio-economic status was defined by education level, based on the highest qualification obtained either in the Netherlands or in the country of origin,^19^ and classified into four categories: no schooling or elementary schooling only, lower vocational schooling or lower secondary schooling, intermediate vocational schooling or intermediate/higher secondary schooling, and higher vocational schooling or university.

#### Ethnicity

Ethnic origin was defined according to participants’ country of birth and that of their parents. Participants were considered of non-Dutch origin if they fulfill either of the following criteria: born abroad and have at least one parent born abroad (first generation), or born in the Netherlands but have both parents born abroad (second generation). After data collection, participants of Surinamese ethnic origin were further classified according to self-reported ethnic origin (obtained by questionnaire), into ‘African’, ‘South-Asian’, ‘Javanese’ and ‘other/unknown’ Surinamese origin. For the Dutch sample, we invited people who were born in the Netherlands and whose parents were born in the Netherlands.

### Statistical analyses

The characteristics of the study population were expressed as numbers (percentages) for categorical variables and as mean±standard deviation (SD) for continuous variables. Descriptive statistical analyses were carried out on characteristics of the participants. Measures of frequencies for the categorical variables and measures of tendency for continuous variables. Normality of the data was tested via histogram and the absolute values of skewness and kurtosis. A chi-square test was applied to examine whether the number of male participants, age and conventional risk factors were significant different between the Dutch and ethnic minorities. For main effects, *P* < 0.05 was considered statistically significant. To investigate whether the associations with age and eGFR differed between sexes when comparing Dutch participants with each group of non-Dutch descent, separate sex-specific regression models were built where eGFR was the dependent variable and age (centered), ethnic background (including Dutch and ethnic group of interest), and the interaction term age * ethnic background were independent variables. Beta coefficients of the last two variables reflect differences in intercept and slope of both regression lines, respectively. For graphical representation non-linear curve fit using GraphPad Prism 8.3.0 was performed.

To further investigate the association of ethnicity (independent variable) with eGFR, multivariable linear regression analyses were carried out using IBM SPSS Statistics 24. Four multivariable models were constructed. Model I represented the age- and sex-adjusted model. Model II additionally included BMI (continuous), hypertension, and diabetes mellitus. In model III, ACR (continuous), smoking and hypercholesterolemia were added. In the final model, model IV, four levels of education as marker for SES were included. To investigate the association of ethnicity with ACR, another four multivariable models were constructed. Again model I represented the age- and sex-adjusted model. Model II was aimed to correct for hyperfiltration, which is associated with higher ACR, by adjusting for BMI (continuous), hypertension, diabetes and eGFR (continuous). In model III, smoking and hypercholesterolemia were added. The final model, model IV, additionally included the four levels of education.

### Role of the funding source

The funders had no role in study design, data collection and analysis, decision to publish, or preparation of the manuscript. B.J.M.V.H. and L.V. had access to the dataset after approval of the data transfer agreement and initiated the submission for publication.

## Results

### Characteristics of study populations

Table 1 shows the baseline characteristics of the population by ethnicity. As published earlier, differences between participants, non-participants and those not contacted were small with regard to sex, age and socioeconomic status within each ethnic group.^17^ Consistent with previous HELIUS publications, there were, however, baseline differences between the groups. Participants of Turkish and Moroccan origin were significantly younger (±5 years) than the other ethnic groups. Hypertension was significantly more prevalent in the participants of South-Asian Surinamese, African Surinamese and Ghanaian origin as compared to participants of Dutch and Turkish origin. Among Moroccan participants a significant lower prevalence of hypertension was observed. Overall, ethnic minority groups were significantly more likely to have obesity, diabetes mellitus, and lower levels of education as compared to participants from Dutch origin. Hypercholesterolemia prevalence was significantly higher in Dutch participants as compared to the other groups. Ghanaian and Moroccan participants were significantly less likely to be smokers as compared to Dutch participants, whereas South-Asian Surinamese, African Surinamese, and Turkish participants smoked significantly more as compared to Dutch participants.

**Table 1:** Baseline characteristics by ethnic origin.

	Dutch (*n* = 4539)	South-Asian Surinamese (*n* = 3027)	African-Surinamese (*n* = 4114)	Ghanaian (*n* = 2297)	Turkish (*n* = 3576)	Moroccan (*n* = 3868)	*P* value
Age, *yr*	46 ± 14	45 ± 13^#‡$^	48 ± 13 ^ʂ*†‡$^	45 ± 11 ^ʂ#‡$^	40 ± 12 ^ʂ*#†^	40 ± 13 ^ʂ*#†^	*P* < 0.001
Male sex,*%*	46	45^#†$^	39 ^ʂ*‡^	39 ^ʂ*‡^	46^# †$^	39 ^ʂ*‡^	*P* < 0.001
BMI, kg/m^2^	24.7 ± 4	26.3 ± 5 ^ʂ#†‡$^	27.8 ± 5.5 ^ʂ*†‡^	28.5 ± 5 ^ʂ*#$^	28.6 ± 6 ^ʂ*#$^	27.6 ± 5 ^ʂ*†‡^	*P* < 0.001
Plasma creatinine, µmol/L^a^	73 (66–83)	71 (61–84) ^ʂ#†‡$^	76 (66–88) ^ʂ*‡$^	77 (66–90) ^ʂ*‡$^	65 (53–76) ^ʂ*#†$^	61 (53–73) ^ʂ*#†‡^	*P* < 0.001
eGFR, mL/min/1.73 m^2^	95 ± 15	97 ± 17 ^ʂ#†‡$^	103 ± 19 ^ʂ*‡$^	104 ± 20 ^ʂ*‡$^	107 ± 14 ^ʂ*#†$^	110 ± 15 ^ʂ*#†‡^	*P* < 0.001
ACR, mg/mmol	0.6 ± 2	2.4 ± 15 ^ʂ#†‡$^	1.2 ± 7 ^ʂ*^	1.4 ± 6.7 ^ʂ*^	1.3 ± 6.9 ^ʂ*^	1.6 ± 12 ^ʂ*^	*P* < 0.001
ACR, mg/mmol^a^	0.25 (0.16–0.40)	0.28 (0.16–0.60)	0.26 (0.16–0.52)	0.25 (0.15–0.53)	0.31 (0.19–0.60)	0.34 (0.19–0.68)	*P* < 0.001
Hypertension,*%*	25	38 ^ʂ #†‡$^	46 ^ʂ*†‡$^	53 ^ʂ *#‡$^	25^*#†$^	19 ^ʂ*#†‡^	*P* < 0.001
Diabetes,*%*	4	22 ^ʂ #†‡$^	14 ^ʂ *‡^	15 ^ʂ *‡^	11 ^ʂ*#†^	12 ^ʂ *^	*P* < 0.001
Smoking,*%*	25	28 ^ʂ #†‡$^	32 ^ʂ*#‡$^	5 ^ʂ*#‡$^	35 ^ʂ *†$^	14 ^ʂ *#†‡^	*P* < 0.001
Hypercholesterolemia*,%*	15	12 ^ʂ†$^	10 ^ʂ*‡$^	11 ^ʂ*#‡$^	9 ^ʂ#†$^	5 ^ʂ*#‡^	*P* < 0.001
First generation,*%*Schooling - None/elementary,*%*	100 3	75.5^#†‡$^ 14 ^ʂ #†‡$^	82.8^*†‡$^ 6^*†‡$^	94.4^*#‡$^ 29 ^ʂ *#‡^	68.7^⁎#†^ 32 ^ʂ*#†^	66.6^⁎#†^ 31 ^ʂ*#^	*P* < 0.001 *P* < 0.001
- Lower vocational or lower secondary,%	14	33 ^ʂ†‡$^	36 ^ʂ †‡$^	40 ^ʂ*#‡$^	25 ^ʂ*#†$^	18 ^ʂ*#†‡^	*P* < 0.001
- Intermediate vocational or intermediate/higher secondary,%	22	29 ^ʂ#†$^	35 ^ʂ*†‡^	25 ^*#$^	29 ^ʂ#$^	33 ^ʂ*†‡^	*P* < 0.001
- Higher vocational or university,%	60	22 ^ʂ †‡$^	23 ^ʂ †‡$^	6 ^ʂ *#‡$^	14 ^ʂ*#†^	17 ^ʂ*#†^	*P* < 0.001

Values for categorical variables are given as number (percentage); for continuous variables, as mean ± standard deviation (except of ^a^, median (IQR)). BMI, body mass index. eGFR, estimated glomerular filtration rate. ACR, urinary albumin-creatinine ratio. Post hoc analysis: ^ʂ^*p* < 0.05 vs Dutch; *p < 0.05 vs South-Asian Surinamese; ^#^*p* < 0.05 vs African Surinamese; ^†^*p* < 0.05 vs Ghanaian; ^‡^*p* < 0.05 vs Turkish; ^$^*p* < 0.05 vs Moroccan.

### Sex-specific associations between age and eGFR

**Figure 1:**
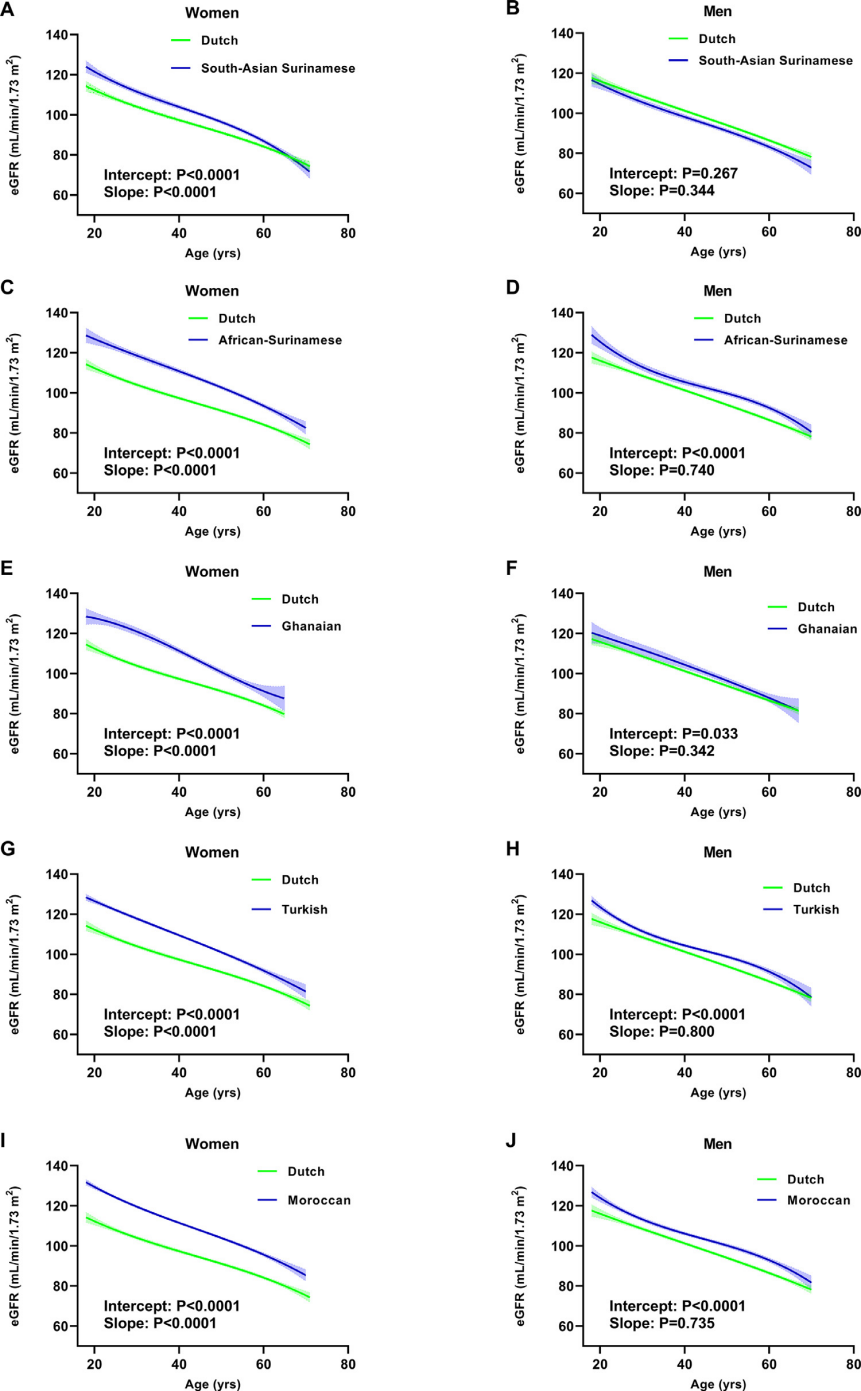
Sex-specific associations between age and eGFR for each ethnic group as compared with Dutch origin participants. Regression lines of age vs. eGFR (non-linear fit with 95% CI). Intercept of each non-Dutch origin ethnicity was significantly higher as compared to Dutch subjects for each ethnic group except for South Asian men. Slopes of each non-Dutch group were significantly different from Dutch participants when comparing women. In men no significant slope differences were observed. CI, confidence interval. eGFR, estimated glomerular filtration rate.

For each ethnic group, except for men of South-Asian Surinamese descent, eGFR was significantly higher as compared to Dutch participants across all ages (Figure 1A–J). Within each minority group, these differences were larger in women as compared to men. In women, the interaction between age and ethnic background was highly significant, reflecting a steeper slope of the regression line for age with eGFR in the ethnic groups as compared to participants of Dutch descent (*p* < 0.001, Figure 1B,D,F,H,J). No slope differences were observed when making comparisons in men (Figure 1B,D, F, H, J).

### Ethnic differences in eGFR

Table 2 shows the results of the multivariable regression analyses on ethnic differences in eGFR. Mean (SD) age and sex-adjusted eGFR was significantly higher in all ethnic minority groups as compared to those of Dutch origin, ranging from 1.5 in South Asian to 10.1 mL/min/1.73 m^2^ in Moroccan participants (*p* < 0.001 for all ethnic minority groups). After adjustment for BMI, hypertension and diabetes mellitus (model II), eGFR differences with Dutch participants modestly diminished in all ethnic groups, except in participants of African Surinamese origin, but remained significantly different (*p* < 0.001). When additionally adjusting for ACR, smoking and non-HDL cholesterol (model III) as well as after adjustment for level of education (model IV), results did not change and again highly significant differences remained (*p* < 0.001).

**Table 2:** Ethnic differences in eGFR: higher levels in all ethnic groups as compared to the Dutch group.

eGFR (mL/min/1.73m^2^)	Model I		Model II		Model III		Model IV	
	Difference (±SE)	P-value	Difference (±SE)	P-value	Difference (±SE)	P-value	Difference (±SE)	P-value
*Dutch(reference)*								
South-Asian	1.5 ± 0.3	<0.001	1.3 ± 0.3	<0.001	1.5 ± 0.3	<0.001	0.9 ± 0.3	<0.001
African Surinamese	9.1 ± 0.3	<0.001	9.1 ± 0.3	<0.001	8.9 ± 0.3	<0.001	8.5 ± 0.3	<0.001
Ghanaian	7.8 ± 0.3	<0.001	8.0 ± 0.3	<0.001	8.2 ± 0.3	<0.001	7.3 ± 0.4	<0.001
Turkish	8.0 ± 0.3	<0.001	7.7 ± 0.3	<0.001	7.6 ± 0.3	<0.001	6.8 ± 0.3	<0.001
Moroccan	10.1 ± 0.3	<0.001	9.8 ± 0.3	<0.001	10.0 ± 0.3	<0.001	9.2 ± 0.3	<0.001

Model I: adjusted for age and sex; Model II: adjusted for age, sex, BMI, hypertension and diabetes mellitus; Model III: adjusted for age, sex, BMI, hypertension, diabetes mellitus, ACR, smoking and plasma non-HDL cholesterol; Model IV: adjusted for age, sex, BMI, hypertension, diabetes mellitus, ACR, smoking, non-HDL cholesterol and level of education.

ACR, urinary albumin-creatinine ratio. BMI, body mass index. HDL, high density lipoprotein. eGFR, estimated glomerular filtration rate.

Because a higher eGFR in the ethnic minority participants may be the result of hyperfiltration due to different prevalence rates in overweight, hypertension or diabetes mellitus,^20^, ^21^, ^22^ a sensitivity analysis was performed to investigate whether presence of overweight (i.e., >25 kg/m^2^), hypertension or diabetes mellitus affected differences in eGFR between ethnic groups. After excluding participants with, respectively, overweight, hypertension, or diabetes, the age- and sex-adjusted eGFR differences as compared to Dutch origin participants remained present to the same extent (Supplemental Tables 1–3). Finally, to assess whether being of the second generation might influence eGFR, we repeated the regression analysis in participants of the first generation only. This analysis showed similar results with significantly higher eGFR values in all ethnic groups as compared to the Dutch group (data not shown).

### Ethnic differences in ACR

Table 3 represents the results of the multivariable regression analyses on ethnic differences in ACR. After consecutive log-transformations, ACR distribution remained similarly skewed (data not shown) and was therefore not transformed for analysis and interpretation to clinical practice. Age- and sex-adjusted mean ACR was significantly higher among all ethnic minority groups as compared to those of Dutch origin. After adjustment for BMI, hypertension, diabetes and eGFR (model II), smoking and non-HDL cholesterol (model III) and level of education (model IV) all ethnic minority groups had a significantly higher ACR as compared to participants of Dutch origin.

**Table 3:** Ethnic differences in ACR: higher levels in all ethnic groups as compared to the Dutch group

ACR (mg/mmol)	Model I		Model II		Model III		Model IV	
	Difference (±SE)	P-value	Difference (±SE)	P-value	Difference (±SE)	P-value	Difference (±SE)	P-value
*Dutch(reference)*								
South-Asian	1.70 ± 0.21	<0.001	1.26 ± 0.21	<0.001	1.25 ± 0.21	<0.001	1.1 ± 0.22	<0.001
African Surinamese	0.46 ± 0.20	0.02	0.79 ± 0.20	<0.001	0.76 ± 0.20	<0.001	0.7 ± 0.21	<0.001
Ghanaian	0.74 ± 0.23	0.001	0.73 ± 0.24	0.002	0.77 ± 0.24	0.001	0.54 ± 0.26	0.008
Turkish	0.84 ± 0.20	<0.001	1.09 ± 0.21	<0.001	1.1 ± 0.21	<0.001	0.8 ± 0.23	<0.001
Moroccan	1.12 ± 0.20	<0.001	1.66 ± 0.21	<0.001	1.7 ± 0.21	<0.001	1.4 ± 0.22	<0.001

Model I: adjusted for age and sex; Model II: adjusted for age, sex, BMI, hypertension, diabetes mellitus and eGFR; Model III: adjusted for age, male sex, BMI, hypertension, diabetes mellitus, eGFR, smoking and plasma non-HDL cholesterol; Model IV: adjusted for age, sex, BMI, hypertension, diabetes mellitus, eGFR, smoking, non-HDL cholesterol and level of education.

ACR, urinary albumin-creatinine ratio. BMI, body mass index. HDL, high density lipoprotein. eGFR, estimated glomerular filtration rate.

A sensitivity analysis (Supplemental Tables 4–6) was performed to assess whether the ethnic differences were still present in participants having no overweight, hypertension or diabetes, respectively. Among individuals without overweight, a significant higher age- and sex-adjusted ACR was observed in all ethnic minority groups compared to Dutch participants, except for Ghanaian participants (Supplemental Table 4). In the fully corrected model (model IV, Supplemental Table 4), ACR differences were also not significant in South-Asian participants. After excluding hypertensive participants, ACR differences were not significant in South-Asian, African Surinamese, and Ghanaian participants (model I, Supplemental Table 5). After full correction (model IV, Supplemental Table 5), no significant differences were observed in the South-Asian Surinamese participants only. Excluding participants with diabetes did not influence the results.

### Ethnic differences in eGFR whilst omitting the ethnicity coefficient

Supplemental Table 7 shows the results of the multivariable analyses on ethnic differences in eGFR when the CKD-EPI equation was used whilst omitting the ethnicity coefficients. The age- and sex-adjusted eGFR in participants from African Surinamese and Ghanaian background was significantly lower, instead of higher, as compared to the Dutch (-4.6 ml/min/1.73 m^2^ and -5.9 ml/min/1.73 m^2^ in participants from, respectively, African Surinamese and Ghanaian descent). Highly significant differences remain, showing lower eGFR after adjustment for BMI, hypertension, diabetes mellitus, ACR, smoking, non-HDL cholesterol and level of education.

## Discussion

In this large multiethnic cohort study, in which six ethnic groups living in Amsterdam are represented, we found a significantly higher eGFR among all ethnic minority groups as compared to participants from Dutch origin. This finding could not be explained by differences in age, sex and risk factors for CKD. After adjustment for traditional cardiovascular and renal risk factors (i.e., BMI, hypertension, diabetes, smoking, hypercholesterolemia) as well as socio-economic status (i.e., low level of education), age- and sex-adjusted eGFR was still significantly higher as compared to participants of Dutch origin, ranging from 1.5 mL/min/1.73 m^2^ in South-Asian Surinamese participants to 10.1 mL/min/1.73 m^2^ in participants of Moroccan origin. When comparing the association between age and eGFR in groups from non-Dutch origin with subjects from Dutch origin, both eGFR and slope of eGFR decline with age was higher in the ethnic minority groups for all ages. Within each minority group, these differences were larger in women as compared to man. Women also showed a more rapid decline of kidney function with age in the ethnic groups as compared to participants of Dutch descent. Exception were men of South-Asian Surinamese descent in whom eGFR was lower as compared to their Dutch counterparts. ACR, reflecting structural damage of kidney, was significantly higher in all ethnic minority groups. This association remained significant after adjustment for multiple variables.

The eGFR findings seem inconsistent with the higher prevalence of CKD in ethnic minorities^6^, ^7^, ^8^ and may imply that eGFR-based screening tools, such as the KDIGO, have important limitations in multiethnic populations.^14^^,^^15^^,^^23^^,^^24^ An alternative interpretation of the higher eGFR in the ethnic minority groups may reflect the presence of hyperfiltration—a biological factor that is considered to be an early sign of microvascular kidney alterations. A higher GFR is thought to be a sign of increased intraglomerular hydrostatic pressure and precedes development of proteinuria, progressive kidney function decline, and overt CKD.^25^^,^^26^ Hyperfiltration is associated with high BMI, hypertension, and diabetes mellitus, and may—given the baseline differences in these parameters between the various ethnic groups in HELIUS—serve as an explanation for the higher eGFR in these groups.^26^^,^^27^ In keeping with this, we found—yet in a cross-sectional fashion—that eGFR declines faster with higher age in women of the ethnic minority groups as compared to Dutch women. However, hyperfiltration is unlikely to explain the difference. First, multiple corrections for baseline differences did not change our eGFR results. Second, sensitivity analyses, in which participants with overweight, hypertension or diabetes mellitus were excluded—i.e., conditions associated with hyperfiltration—did not alter the eGFR differences between Dutch and ethnic minority groups. Third, the concept that hyperfiltration represents an early sign of structural kidney changes leading to CKD has been based on true (measured) GFR, whereas the use serum creatinine-based estimations—including eGFR according to CKD-EPI equation—cannot be used for the purpose of identifying hyperfiltration.^25^^,^^28^

Since the publication of the MDRD equation, and later on the CKD-EPI equation, it has been acknowledged that biological non-GFR related differences associated with ethnic background may affect eGFR. Due to differences in muscle mass between Europeans, Asians and African Americans, usually leading to higher serum creatinine concentrations in African Americans, the creatinine-based CKD-EPI equation includes a correction factor.^11^ We used the same correction factor in our cohort for subjects from African descent (i.e., Ghanaians and African Surinamese participants). Omitting the ethnicity coefficient from the CKD-EPI equation leads, however, to a significant lower, not higher, eGFR in participants from African Surinamese and Ghanaian origin, also after multiple adjustments. This finding supports the notion that inclusion of the ethnicity coefficient leads to overestimating eGFR in those groups. In keeping with this, a very recent interim report of the NKF-ASN task force has =questioned the use of an ethnicity coefficient in calculation of the eGFR, not only because this correction will not capture all biological factors that explain differences between subjects from African and non-African descent.^29^ It may also underappreciate ancestral diversity among people of African descent and may even obscure disparities in health and healthcare.^29^ A validation study comparing measured GFR with MDRD eGFR in South Africans of African descent found, for example, that the use of the ethnic correction factor resulted in a median positive bias of 13 mL/min/1.73 m^2^ whereas no bias was observed without correcting.^24^ More recently, comparison between pair-matched African Europeans and Europeans also demonstrated a positive bias of CKD-EPI eGFR in the African population,^14^ whereas in African Americans having CKD omission of race in this equation resulted in more underestimation of the GFR.^15^ In South Asians living in Pakistan, it was found that CKD-EPI eGFR overestimates true GFR.^23^ Data with regard to the other ethnic groups included our cohort remain limitedly reported. Furthermore, it is not known whether subjects from African and South Asian origin living in other continents resemble their counterparts living in Europe with regard to non-GFR determinants. In this respect, also the Dutch, Turkish or Moroccan HELIUS participants may not resemble the subjects from European descent included in the large US-based cohorts. The discrepancies in eGFR between the HELIUS groups may, therefore, not reflect information on true GFR, i.e., the parameter of interest in predicting CKD-related outcomes, and eGFR use in our cohort may not correctly identify patients at risk for CKD. This is in line with previously observed higher CKD prevalence and the more frequent presence of CKD risk factors in the non-Dutch ethnic groups of the HELIUS cohort.^6^^,^^19^

Our findings regarding the higher ACR in the non-Dutch groups are more in line with previously demonstrated higher prevalence of CKD in ethnic minority groups. Higher ACR already within the range normoalbuminuria, has been recognized as risk factor for development of CKD independent of eGFR.^4^^,^^30^ Regarding the (presumably spurious) high eGFR values that we found in the non-Dutch ethnic groups, ACR may prove to serve as better future predictor for CKD-related events in a multiethnic population. ACR, however, depends on both absolute urine albumin concentration and creatinine excretion in the urine. Since urine creatinine excretion may also vary among various ethnic groups, amongst others due to differences in muscle mass,^31^ ACR might either over or underestimate CKD risk in some ethnic minority groups, in whom creatinine generation may not be the same, and may consequently lose some of its predictive value.

Based on our study results, adoption of the currently recommended approach for staging and predicting CKD and CKD-related complications, by using the composite of both eGFR and ACR, potentially leads to uncertainty in CKD prevalence and incorrect estimation of the risk of CKD-related complications in a multi-ethnic population. Particularly, in ethnic groups that are recognized for a higher risk of CKD, eGFR is higher while ACR may be underestimated (due to higher creatinine generation) as compared to ethnic groups with a low risk of CKD. On one hand our findings prompt for exploration of other risk factors that explain the higher CKD prevalence in certain ethnic groups—e.g., genetic predisposition, including the presence of APOL1 gene variants, the biology of creatinine excretion, dietary variations and cultural differences. On the other hand, our data—in line with others, including the recent NKF-ASN taskforce—underscore the need to reassess the value of ethnic specific tools for predicting CKD risk and long-term CKD-related outcome.^12^^,^^13^^,^^29^^,^^32^

Our study has several strengths. We report data from a very large sample from a European general population, in which six ethnic groups are represented in a balanced way. We acknowledge some limitations as well. As data consist of single measurement of eGFR and albuminuria inferences on diagnosing CKD may be overestimated as this requires the presence of low eGFR or albuminuria for at least 3 months, which makes it difficult to determine any major conclusions regarding their predictive value. Follow-up data will become available within the coming years, as currently all participants are invited for follow-up visits. Furthermore, due to inclusion of over 20,000 participants and resource limitations, we were not able to validate eGFR against measured GFR, as has been done in the studies that validated the MDRD and CKD-EPI equations.^33^ So the question whether differences between the groups are caused by differences in true GFR or errors in its estimation by using serum creatinine cannot be completely answered in our dataset. Nevertheless, we were able to quantify disparities in eGFR and ACR levels among 6 ethnic groups with adjustments for the most frequently reported factors driving disparities in CKD risk. As such, these data can be easily translated to practice where both eGFR and ACR are used for screening for CKD. Cystatin C was not measured in order to allow eGFR calculation based on both serum creatinine and cystatin C. This eGFR equation appears to be less influenced by non-GFR determinants—e.g., age, sex, and muscle mass, particularly when eGFR is >60 mL/min/1.73 m^2^.^15^ It is however unknown that inclusion of cystatin C will completely annul the discrepancies for each ethnic group.^23^

To summarize, in this multiethnic cohort study, including large representative samples of six ethnic groups from Amsterdam, the Netherlands, we found clinically significant discrepancies as compared to Dutch participants in both eGFR, ranging from 1.5 ± 0.30 in South-Asian Surinamese to 10.1 ± 0.28 mL/min/1.73 m^2^ in Moroccan participants, and ACR, ranging from 0.46 ± 0.20 in African Surinamese participants to 1.70 ± 0.21 mg/mmol in South-Asian Surinamese participants, between participants of Dutch origin and their counterparts of non-Dutch origin. These discrepancies could not be attributed to differences in factors that may affect kidney function or influence the risk of kidney damage. Our findings might have implications for the currently recommended approach for staging and predicting CKD and CKD-related complications in multiethnic populations and deserves further exploration.

## Data sharing statement

The HELIUS study has an open policy in regard to collaboration with other research groups. Before access to data is provided, all researchers should carefully read and must adhere to the HELIUS Collaboration Policy (https://www.heliusstudy.nl/nl/researchers/collaboration/).

## Author contributions

R.J.G.P., M.B.S. and L.V. designed the analysis plan; B.J.M.V.H., M.B.S. and L.V. carried out the statistical analyses; B.J.M.V.H., C.A., B.J.H.vd.B, L.V. drafted and revised the manuscript; all authors approved the final version of the manuscript.

## Funding

The HELIUS study is conducted by the Amsterdam University Medical Center and the Public Health Service of Amsterdam. Both organizations provided core support for HELIUS. The HELIUS study is also funded by the Dutch Heart Foundation (2010T084), the Netherlands Organization for Health Research and Development (ZonMw:200500003), the European Union (FP-7: 278901), and the European Fund for the Integration of non-EU immigrants (EIF:2013EIF013).

## Declaration of interests

The authors declare that they have no relevant financial interests.

## Supplementary materials

Image, application 1
