# Supplementary material for: Discrepancies in estimated glomerular filtration rate and albuminuria levels in ethnic minority groups – The multiethnic HELIUS cohort study
Source: eClinicalMedicine. 2022 Mar 5;45:101324. doi: 10.1016/j.eclinm.2022.101324 (PMC8904239; doi:10.1016/j.eclinm.2022.101324)
Supplement: Supplementary file 1 [file mmc1.docx]

**SUPPLEMENTAL DATA to Huisman BJMV et al.**

**TABLE S1**.

*Title:* Sensitivity analysis in non-overweight participants, dependent eGFR

*Note*: Model I: adjusted for age and sex; Model II: adjusted for age, sex, hypertension and diabetes; Model III: adjusted for age, sex, hypertension, diabetes, ACR, smoking and plasma non-HDL cholesterol; Model IV: adjusted for age, sex, hypertension, diabetes, ACR, plasma non-HDL cholesterol and level of education.

**TABLE S2**.

*Title:* Sensitivity analysis in normotensive participants, dependent eGFR

*Note*: Model I: adjusted for age and sex; Model II: adjusted for age, sex, BMI and diabetes; Model III: adjusted for age, sex, BMI, diabetes, ACR, smoking and plasma non-HDL cholesterol; Model IV: adjusted for age, sex, BMI, diabetes, ACR, plasma non-HDL cholesterol and level of education.

**TABLE S3**.

*Title:* Sensitivity analysis in participants without diabetes, dependent eGFR

*Note*: Model I: adjusted for age and sex; Model II: adjusted for age, sex, BMI and hypertension; Model III: adjusted for age, sex, BMI, hypertension, ACR, smoking and plasma non-HDL cholesterol; Model IV: adjusted for age, sex, BMI, hypertension, ACR, plasma non-HDL cholesterol and level of education.

**TABLE S4**.

*Title:* Sensitivity analysis in non-overweight participants, dependent ACR

*Note*: Model I: adjusted for age and sex; Model II: adjusted for age, sex, hypertension, diabetes and eGFR; Model III: adjusted for age, sex, hypertension, diabetes, eGFR, smoking and plasma non-HDL cholesterol; Model IV: adjusted for age, sex, hypertension, diabetes, eGFR, plasma non-HDL cholesterol and level of education.

**TABLE S5.**

*Title:* Sensitivity analysis in normotensive participants, dependent ACR

*Note*: Model I: adjusted for age and sex; Model II: adjusted for age, sex, BMI, diabetes and eGFR; Model III: adjusted for age, sex, BMI, diabetes, eGFR, smoking and plasma non-HDL cholesterol; Model IV: adjusted for age, sex, BMI, diabetes, eGFR, plasma non-HDL cholesterol and level of education.

**TABLE S6**.

*Title:* Sensitivity analysis in participants without diabetes, dependent ACR

*Note*: Model I: adjusted for age and sex; Model II: adjusted for age, sex, BMI, hypertension and eGFR; Model III: adjusted for age, sex, BMI, hypertension, eGFR, smoking and plasma non-HDL cholesterol; Model IV: adjusted for age, sex, BMI, hypertension, eGFR, plasma non-HDL cholesterol and level of education.

**TABLE S7.**

*Title:* Ethnic differences in eGFR : using the CKD-EPI equation without the race/ethnicity correction

*Note:* Model I: adjusted for age and sex; Model II: adjusted for age, sex, BMI, hypertension and diabetes mellitus; Model III: adjusted for age, sex, BMI, hypertension, diabetes mellitus, ACR, smoking and plasma non-HDL cholesterol; Model IV: adjusted for age, sex, BMI, hypertension, diabetes mellitus, ACR, smoking, non-HDL cholesterol and level of education. ACR, urinary albumin-creatinine ratio. BMI, body mass index. HDL, high density lipoprotein. eGFR, estimated glomerular filtration rate, without the race/ethnicity correction.

**Table S1. Sensitivity analysis in non-overweight participants, dependent eGFR**

|  | **Model I** | | **Model II** | | **Model III** | | **Model IV** | |
| --- | --- | --- | --- | --- | --- | --- | --- | --- |
|  |  |  |  |  |  |  |  |  |
|  |  |  |  |  |  |  |  |  |
| eGFR (ml/min/1.73m^2^) | Difference (±SE) | P-value | Difference (±SE) | P-value | Difference (±SE) | P-value | Difference (±SE) | P-value |
|  |  |  |  |  |  |  |  |  |
| *Dutch(reference)* |  |  |  |  |  |  |  |  |
| South-Asian | 1.5 ± 0.42 | <0.001 | 1.4 ± 0.42 | 0.001 | 1.5 ± 0.43 | <0.001 | 1.4 ± 0.43 | 0.001 |
| African Surinamese | 10.6 ± 0.41 | <0.001 | 10.8 ± 0.41 | <0.001 | 10.5 ± 0.42 | <0.001 | 10.5 ± 0.42 | <0.001 |
| Ghanaian | 9.3 ± 0.58 | <0.001 | 9.6 ± 0.58 | <0.001 | 9.7 ± 0.58 | <0.001 | 9.4 ± 0.59 | <0.001 |
| Turkish | 8.5 ± 0.47 | <0.001 | 8.5 ± 0.47 | <0.001 | 8.4 ± 0.48 | <0.001 | 8.2 ± 0.48 | <0.001 |
| Moroccan | 10.4 ± 0.43 | <0.001 | 10.4 ± 0.43 | <0.001 | 10.5 ± 0.43 | <0.001 | 10.2 ± 0.44 | <0.001 |
|  |  |  |  |  |  |  |  |  |

**Table S2. Sensitivity analysis in normotensive participants, dependent eGFR**

|  | **Model I** | | **Model II** | | **Model III** | | **Model IV** | |
| --- | --- | --- | --- | --- | --- | --- | --- | --- |
|  |  |  |  |  |  |  |  |  |
|  |  |  |  |  |  |  |  |  |
| eGFR (ml/min/1.73m^2^) | Difference (±SE) | P-value | Difference (±SE) | P-value | Difference (±SE) | P-value | Difference (±SE) | P-value |
|  |  |  |  |  |  |  |  |  |
| *Dutch(reference)* |  |  |  |  |  |  |  |  |
| South-Asian | 2.4 ± 0.34 | <0.001 | 2.1 ± 0.35 | <0.001 | 2.1 ± 0.35 | <0.001 | 2.0 ± 0.35 | <0.001 |
| African Surinamese | 10.2 ± 0.32 | <0.001 | 10.1 ± 0.33 | <0.001 | 9.8 ± 0.33 | <0.001 | 9.9 ± 0.33 | <0.001 |
| Ghanaian | 9.4 ± 0.42 | <0.001 | 9.2 ± 0.42 | <0.001 | 9.4 ± 0.43 | <0.001 | 9.1 ± 0.43 | <0.001 |
| Turkish | 8.4 ± 0.31 | <0.001 | 8.2 ± 0.32 | <0.001 | 8.1 ± 0.32 | <0.001 | 7.7 ± 0.33 | <0.001 |
| Moroccan | 10.6 ± 0.3 | <0.001 | 10.4 ± 0.31 | <0.001 | 10.4 ± 0.31 | <0.001 | 10.1 ± 0.31 | <0.001 |
|  |  |  |  |  |  |  |  |  |

**Table S3. Sensitivity analysis in participants without diabetes, dependent eGFR**

|  | **Model I** | | **Model II** | | **Model III** | | **Model IV** | |
| --- | --- | --- | --- | --- | --- | --- | --- | --- |
|  |  |  |  |  |  |  |  |  |
|  |  |  |  |  |  |  |  |  |
| eGFR (ml/min/1.73m^2^) | Difference (±SE) | P-value | Difference (±SE) | P-value | Difference (±SE) | P-value | Difference (±SE) | P-value |
|  |  |  |  |  |  |  |  |  |
| *Dutch(reference)* |  |  |  |  |  |  |  |  |
| South-Asian | 1.5 ± 0.32 | <0.001 | 1.7 ± 0.32 | <0.001 | 1.7 ± 0.32 | <0.001 | 1.6 ± 0.32 | <0.001 |
| African Surinamese | 8.8 ± 0.29 | <0.001 | 9.0 ± 0.29 | <0.001 | 8.8 ± 0.29 | <0.001 | 8.8 ± 0.29 | <0.001 |
| Ghanaian | 7.7 ± 0.35 | <0.001 | 8.1 ± 0.35 | <0.001 | 8.2 ± 0.35 | <0.001 | 7.9 ± 0.36 | <0.001 |
| Turkish | 7.7 ± 0.31 | <0.001 | 7.7 ± 0.31 | <0.001 | 7.6 ± 0.31 | <0.001 | 7.3 ± 0.32 | <0.001 |
| Moroccan | 9.9 ± 0.30 | <0.001 | 9.9 ± 0.30 | <0.001 | 10.1 ± 0.30 | <0.001 | 9.7 ± 0.31 | <0.001 |
|  |  |  |  |  |  |  |  |  |

**Table S4. Sensitivity analysis in non-overweight participants, dependent ACR**

|  | **Model I** | | **Model II** | | **Model III** | | **Model IV** | |
| --- | --- | --- | --- | --- | --- | --- | --- | --- |
|  |  |  |  |  |  |  |  |  |
|  |  |  |  |  |  |  |  |  |
| ACR (mg/mmol) | Difference (±SE) | P-value | Difference (±SE) | P-value | Difference (±SE) | P-value | Difference (±SE) | P-value |
|  |  |  |  |  |  |  |  |  |
| *Dutch(reference)* |  |  |  |  |  |  |  |  |
| South-Asian | 0.58 ± 0.22 | 0.009 | 0.40 ± 0.23 | 0.087 | 0.39 ± 0.23 | 0.092 | 0.35 ± 0.23 | 0.127 |
| African Surinamese | 0.47 ± 0.22 | 0.033 | 0.55 ± 0.23 | 0.016 | 0.53 ± 0.23 | 0.022 | 0.53 ± 0.23 | 0.022 |
| Ghanaian | 0.48 ± 0.31 | 0.118 | 0.42 ± 0.32 | 0.188 | 0.44 ± 0.32 | 0.165 | 0.36 ± 0.32 | 0.268 |
| Turkish | 0.84 ± 0.25 | 0.001 | 0.96 ± 0.26 | <0.001 | 0.94 ± 0.26 | <0.001 | 0.87 ± 0.26 | 0.001 |
| Moroccan | 1.2 ± 0.23 | <0.001 | 1.35 ± 0.24 | <0.001 | 1.36 ± 0.24 | <0.001 | 1.3 ± 0.24 | <0.001 |
|  |  |  |  |  |  |  |  |  |

**Table S5. Sensitivity analysis in normotensive participants, dependent ACR**

|  | **Model I** | | **Model II** | | **Model III** | | **Model IV** | |
| --- | --- | --- | --- | --- | --- | --- | --- | --- |
|  |  |  |  |  |  |  |  |  |
|  |  |  |  |  |  |  |  |  |
| ACR (mg/mmol) | Difference (±SE) | P-value | Difference (±SE) | P-value | Difference (±SE) | P-value | Difference (±SE) | P-value |
|  |  |  |  |  |  |  |  |  |
| *Dutch(reference)* |  |  |  |  |  |  |  |  |
| South-Asian | 0.24 ± 0.15 | 0.099 | 0.24 ± 0.15 | 0.107 | 0.22 ± 0.15 | 0.144 | 0.22 ± 0.15 | 0.145 |
| African Surinamese | 0.22 ± 0.14 | 0.123 | 0.29 ± 0.15 | 0.048 | 0.30 ± 0.15 | 0.039 | 0.30 ± 0.15 | 0.039 |
| Ghanaian | 0.29 ± 0.18 | 0.112 | 0.36 ± 0.19 | 0.057 | 0.37 ± 0.19 | 0.048 | 0.37 ± 0.19 | 0.051 |
| Turkish | 0.41 ± 0.13 | 0.002 | 0.47 ± 0.14 | 0.001 | 0.46 ± 0.14 | 0.001 | 0.46 ± 0.15 | 0.002 |
| Moroccan | 0.48 ± 0.13 | <0.001 | 0.55 ± 0.14 | <0.001 | 0.56 ± 0.14 | <0.001 | 0.56 ± 0.14 | <0.001 |
|  |  |  |  |  |  |  |  |  |

**Table S6. Sensitivity analysis in participants without diabetes, dependent ACR**

|  | **Model I** | | **Model II** | | **Model III** | | **Model IV** | |
| --- | --- | --- | --- | --- | --- | --- | --- | --- |
|  |  |  |  |  |  |  |  |  |
|  |  |  |  |  |  |  |  |  |
| ACR (mg/mmol) | Difference (±SE) | P-value | Difference (±SE) | P-value | Difference (±SE) | P-value | Difference (±SE) | P-value |
|  |  |  |  |  |  |  |  |  |
| *Dutch(reference)* |  |  |  |  |  |  |  |  |
| South-Asian | 0.43 ± 0.17 | 0.013 | 0.39 ± 0.17 | 0.022 | 0.40 ± 0.17 | 0.022 | 0.38 ± 0.18 | 0.032 |
| African Surinamese | 0.29 ± 0.15 | 0.053 | 0.49 ± 0.16 | 0.002 | 0.47 ± 0.16 | 0.003 | 0.48 ± 0.16 | 0.003 |
| Ghanaian | 0.67 ± 0.19 | <0.001 | 0.70 ± 0.19 | <0.001 | 0.72 ± 0.19 | <0.001 | 0.66 ± 0.20 | 0.001 |
| Turkish | 0.52 ± 0.16 | 0.001 | 0.76 ± 0.17 | <0.001 | 0.74 ± 0.17 | <0.001 | 0.68 ± 0.17 | <0.001 |
| Moroccan | 0.68 ± 0.16 | <0.001 | 1.10 ± 0.16 | <0.001 | 1.10 ± 0.17 | <0.001 | 1.02 ± 0.17 | <0.001 |
|  |  |  |  |  |  |  |  |  |

**Table S7. Ethnic differences in eGFR : using the CKD-EPI equation without the race/ethnicity correction**

|  | **Model I** | | **Model II** | | **Model III** | | **Model IV** | |
| --- | --- | --- | --- | --- | --- | --- | --- | --- |
|  |  |  |  |  |  |  |  |  |
|  |  |  |  |  |  |  |  |  |
| eGFR (mL/min/1.73m^2^) | Difference (±SE) | P-value | Difference (±SE) | P-value | Difference (±SE) | P-value | Difference (±SE) | P-value |
|  |  |  |  |  |  |  |  |  |
| *Dutch(reference)* |  |  |  |  |  |  |  |  |
| South-Asian | 1.5 ± 0.3 | <0.001 | 1.3 ± 0.3 | <0.001 | 1.5 ± 0.3 | <0.001 | 0.9 ± 0.3 | <0.001 |
| African Surinamese | -4.6 ± 0.3 | <0.001 | -4.6 ± 0.3 | <0.001 | -4.8 ± 0.3 | <0.001 | -4.8 ± 0.3 | <0.001 |
| Ghanaian | -5.9 ± 0.3 | <0.001 | -5.8 ± 0.3 | <0.001 | -5.6 ± 0.3 | <0.001 | -5.8 ± 0.3 | <0.001 |
| Turkish | 8.0 ± 0.3 | <0.001 | 7.7 ± 0.3 | <0.001 | 7.6 ± 0.3 | <0.001 | 6.8 ± 0.3 | <0.001 |
| Moroccan | 10.1 ± 0.3 | <0.001 | 9.8 ± 0.3 | <0.001 | 10.0 ± 0.3 | <0.001 | 9.2 ± 0.3 | <0.001 |
|  |  |  |  |  |  |  |  |  |
